# New Insights Into Factors Shaping CMV‐Specific T‐Cell Polyfunctionality After Hematopoietic Cell Transplantation

**DOI:** 10.1002/ajh.70152

**Published:** 2025-12-01

**Authors:** Alicja Sadowska‐Klasa, Fang Yun Lim, Hu Xie, Danniel Zamora, Terry Stevens‐Ayers, Wendy M. Leisenring, Bradley C. Edmison, Stephen C. De Rosa, Marco Mielcarek, Michael Boeckh

**Affiliations:** ^1^ Fred Hutchinson Cancer Center Seattle Washington USA; ^2^ Medical University of Gdansk Gdansk Poland; ^3^ University of Washington Seattle Washington USA

**Keywords:** CMV‐specific T cells, drug effects, HCT, immunosuppressive treatment

## Abstract

Polyfunctional cytomegalovirus (CMV)‐specific T cells are critical for antiviral immunity in allogeneic hematopoietic cell transplantation (HCT) recipients. However, gaps remain in managing refractory CMV and optimizing virus‐specific cellular therapy (VST). We conducted a comparative analysis of how timing and dosing of immunosuppressive agents, including post‐transplant cyclophosphamide (PT‐Cy), corticosteroids, mycophenolate mofetil (MMF), and calcineurin inhibitors (CNIs), shape CMV‐specific T‐cell polyfunctionality. CD4^+^ and CD8^+^ T‐cell responses (IFN‐γ plus ≥ 1 functional marker) were assessed following pp65 stimulation within 100 days post‐HCT in the pre‐ and post‐letermovir era. Among 243 patients, 31% exhibited polyfunctional CD4^+^ and CD8^+^ responses. PT‐Cy did not significantly impair T‐cell functionality. Cyclosporine was associated with higher frequencies of polyfunctional T cells compared to tacrolimus, even at concentrations above the therapeutic range. Intermediate (≥ 0.5 mg/kg) and high‐dose (≥ 1 mg/kg) corticosteroids, especially within 2–4 weeks before testing, significantly suppressed T‐cell responses, though rapid tapering preserved function. Prolonged administration of MMF (≥ 2000 mg) diminished T‐cell polyfunctionality. These findings provide novel insights into the importance of timing and dosing of the immunosuppressive effects of PT‐Cy, corticosteroids, MMF and CNIs on CMV‐specific immunity. Adjusting immunosuppression in specific time windows may improve the management of refractory CMV infections and optimize VST in HCT recipients.

## 1 Introduction

Cytomegalovirus (CMV) specific T‐cell responses play an important role in controlling CMV infection in allogeneic hematopoietic cell transplant (HCT) recipients, and current clinical strategies increasingly incorporate cell‐mediated immunity into monitoring protocols [1, 2, 3, 4]. In addition to interferon gamma (IFN‐γ), polyfunctional T‐cells produce other cytokines and may offer stronger antiviral protection [5, 6]. As CMV complications continue to occur [7, 8, 9, 10], a deeper understanding of the factors influencing CMV‐specific T‐cell polyfunctionality is critical for managing refractory or resistant CMV infections and for guiding the design of T‐cell therapy trials, especially as modern transplant approaches increase the risk of viral complications [9].

Transplant recipients face severe immune modulation from a range of treatments and prophylaxis, affecting cell‐mediated immunity and protection from novel and latent infections in a myriad of ways. Corticosteroids are a known suppressor of T‐cell immunity, but existing data are based on outdated assays and do not account for other commonly used immunosuppressants [11]. Although clinical practice now favors lower corticosteroid doses [12, 13], the effects of these moderate exposures on CMV‐specific immunity remain poorly characterized. Similarly, the impact of other agents such as mycophenolate mofetil (MMF), calcineurin inhibitors (CNIs), and post‐transplant cyclophosphamide (PT‐Cy) on antiviral T‐cell function is not well understood. Insights from solid organ transplantation (SOT) suggest that different immunosuppressive drugs variably affect control of latent infections, such as BK reactivation, but these findings may not directly translate to HCT settings [14, 15, 16, 17, 18]. Indeed, while reducing immunosuppression for severe infections is well established in the management of SOT recipients [19], it is less frequently employed in the HCT population.

Recent studies demonstrate high efficacy of third‐party or donor‐derived virus‐specific T‐cells (VST) in treating refractory or resistant CMV infection; however, the importance of immune suppression via systemic corticosteroids may diminish the expansion of transfused cells and reduce the in vivo antiviral response [20, 21]. Indeed, clinical trials of CMV‐directed cellular immunotherapy have typically excluded patients treated with systemic steroids of varying doses (Table S1), though these individuals have the greatest need for VST therapy. Also, the effects of other immunosuppressive agents remain insufficiently studied, and small cohort sizes limit the ability to identify drug‐specific effects, and there is limited guidance on how to consider dosing and timing of these agents in VST protocols.

Given the complexity of CMV‐specific immune reconstitution, shaped by immunosuppressive regimens, prophylaxis, viral dynamics, and host factors, there is a pressing need for more granular analysis. This study aims to address that gap by evaluating how corticosteroid use (including dose and timing), exposure to MMF and calcineurin inhibitors, PT‐Cy, and CMV reactivation influence polyfunctional CMV‐specific T‐cell responses after HCT. We applied stratified drug exposure thresholds to capture the nuanced effects of immunosuppression. Additionally, we analyzed combinations of immunosuppressive agents and tracked immune recovery over time to better understand how these variables interact to shape antiviral immunity.

## 2 Methods

This study included CMV seropositive HCT recipients who underwent allogeneic HCT at Fred Hutchinson Cancer Center (Fred Hutch) with available data regarding immune function tests from eras of preemptive therapy (2001–2017), and letermovir (2018–2020). Before 2018, high‐dose valacyclovir for CMV prophylaxis was routinely administered in all cord blood graft recipients [22]. Letermovir prophylaxis was initiated per institutional standard practice at day 8 after high‐risk non–cord HCT, day 1 after high‐risk cord HCT, or engraftment after low‐risk HCT as previously described [23].

All patients underwent weekly CMV DNA PCR surveillance in the first 100 days after HCT with a PCR assay developed at the University of Washington (before November 2019) with a limit of detection (LoD) and limit of quantitation (LoQ) of 7.5 and 25 IU/mL, respectively, and later with the Abbott RealTime Assay with LoD 31.2 IU/mL and LoQ 50 IU/mL. Management of CMV reactivation before day 100 varied slightly over time as was previously described [24, 25].

Peripheral blood mononuclear cell (PBMC) isolation and intracellular cytokine staining for functional CMV‐specific T‐cell markers after pp65 antigen stimulation were carried out as previously described [24, 26, 27]. DMSO background and Staphylococcus enterotoxin B (SEB) positive control stimulations were also included. In a subset of patients, additional stimulation with Immediate Early protein 1 (IE‐1) was performed. All samples were assayed for four core functional immune markers (IFN‐γ, CD107a, TNFα, IL‐2). Samples were collected close to day 90 after transplantation. For a subset of the study group, longitudinal samples collected on day 30, 60 and 90 after HCT were available for additional analyses.

Samples were obtained from previously published studies [24, 28] as well as from an ongoing prospective surveillance protocol conducted at Fred Hutch. For the clinical trial component samples [28], only patients enrolled at Fred Hutch were included, as detailed chart review was integral to this analysis.

### 2.1 Flow Cytometry and Analysis

Detailed laboratory methods, including reagents, stimulation protocols, and flow cytometry procedures, are provided in the Data S1.

Polyfunctional CMV‐specific T‐cell subsets were defined as those expressing IFN‐γ plus ≥ 1 functional marker (CD107a, TNFα or IL‐2) with a frequency greater than 0.05% after background subtraction and at least threefold greater than DMSO response within the same cell population [29]. A CMV‐specific T response with a potential to control CMV infection was defined as an absolute polyfunctional cell count of ≥ 0.5 cell/μL [30]. Unless otherwise noted, any CMV‐specific T‐cell counts in this manuscript are shown with background subtracted and refer to polyfunctional T‐cells as defined above.

### 2.2 Clinical Data

Data on the use of MMF and corticosteroids on the day of sample collection and within 2 and 4 weeks of collection for the immune assays were obtained from medical records. Weight–based doses of prednisone‐equivalent steroid (milligrams per kilogram), were categorized into three groups: low (> 0 to < 0.5 mg/kg), intermediate (≥ 0.5 to < 1 mg/kg), or high (≥ 1 mg/kg). MMF dose was classified as low (< 2000 mg) and high (≥ 2000 mg).

### 2.3 Statistical Analysis

The primary endpoint of this study was the recovery of polyfunctional CMV‐specific CD4^+^ and CD8^+^ responses at 3 months post‐transplantation. Absolute lymphocyte counts (ALCs) drawn at approximately day 30, 60 and 90 after HCT were used to calculate absolute polyfunctional CMV‐specific T‐cell counts (cells per μL) for corresponding time points. Absolute counts of polyfunctional CMV‐specific CD4^+^ and CD8^+^ T‐cell responses were analyzed separately, as both subsets have been implicated in protection against CMV reactivation. Wilcoxon rank‐sum test and Fisher's exact test were used for comparisons of absolute polyfunctional T‐cell counts, as appropriate.

Univariable and multivariable logistic and linear regression models were used to evaluate associations between risk factors and CMV‐specific T‐cell responses and polyfunctionality levels, respectively. Variables with *p* ≤ 0.2 in univariable analysis were candidates for inclusion in the multivariable models. Covariates included in the final MV models were CS and MMF doses, letermovir administration, and CMV viremia status.

To visualize longitudinal changes in CD4^+^ and CD8^+^ count over time, smoothed conditional mean plots were generated in R Statistical Software. Generalized additive mixed (GAM) models were used to visualize CD4^+^ and CD8^+^ responses with respect to PT‐Cy use [31, 32]. The analysis integrated subject‐specific random intercepts to accommodate potential within‐subject correlation, and smooth functions of time using a sample's collection date relative to the individual's HCT date. These random intercepts are assumed to follow a normal distribution. For GAM models, thin plate regression splines with 3 basis dimensions were carried out using the mgcv R package.

Two‐sided *p* values < 0.05 were considered statistically significant. GAM analyses were conducted using R Statistical Software (v4.2.1, R Core Team 2022). All other statistical analyses were performed using SAS 9.4 TS1M6 for Windows (SAS Institute, Cary, NC).

## 3 Results

Samples from 243 CMV seropositive patients collected on day 90 post‐HCT were analyzed in this study. All patients had flow cytometry results following pp65 stimulation. In a subset of patients (*n* = 140), additional results after IE‐1 stimulation were available, which permitted the analysis in selected subsets. Additional samples collected on day 30 and 60 post‐HCT were available in 81 and 77 patients, respectively, were used for a longitudinal assessment. The basic demographic characteristics are summarized in Table 1 (day 90 samples) and Table S2 (day 30, day 60 samples).

**TABLE 1:** Basic demographics of the study group.

Variables	Categories	Total (*N* = 243)
Age at transplant	Median (Range)	52.4 (16.1–74.1)
Sex	Female	101 (42%)
	Male	142 (58%)
Underlying disease	AML/MDS	128 (53%)
	ALL	23 (9%)
	Chronic Lymphoproliferative	56 (23%)
	Chronic myeloid	36 (15%)
Cell source	PBSC	201 (83%)
	Cord Blood	13 (5%)
	Bone Marrow	29 (12%)
Donor CMV status	Negative	146 (60%)
	Positive	97 (40%)
HLA	Related matched	80 (33%)
	Related mismatched (including haplo)	20 (8%)
	Cord Blood	13 (5%)
	Unrelated matched	109 (45%)
	Unrelated mismatched	21 (9%)
TCD or ATG or campath (alemtuzumab)	No	231 (95%)
	Yes	12 (5%)
Conditioning regimen	Myeloablative	154 (63%)
	Non‐myeloablative	89 (37%)
GvHD prophylaxis	CNI + MMF	76 (31%)
	CNI + MTX	112 (46%)
	PT‐Cy	33 (14%)
	Sirolimus based	17 (7%)
	Other	5 (2%)
Acute GvHD	Grade 0–2	208 (86%)
	Grade 3–4	35 (14%)
Chronic GvHD	No	91 (37%)
	Yes	152 (63%)
MMF 0–14 days prior to sample collection d90	No	191 (79%)
	Yes	52 (21%)
MMF exposure days	No MMF	134 (55%)
	0–35 days	32 (13%)
	> 35 days	77 (32%)
MMF dosage	None	192 (79%)
	Low (< 2000 mg)	24 (10%)
	High (≥ 2000 mg)	27 (11%)
Steroid on day 90	None	145 (60%)
	> 0 to < 0.5 mg/kg	51 (21%)
	≥ 0.5 to < 1 mg/kg	28 (12%)
	≥ 1 mg/kg	19 (8%)
Maximum steroid within 0–2 weeks before sample collection	None	112 (46%)
	> 0 to < 0.5 mg/kg	64 (26%)
	≥ 0.5 to < 1 mg/kg	29 (12%)
	≥ 1 mg/kg	38 (16%)
Maximum steroid within 2–4 weeks before sample collection	None	95 (39%)
	> 0 to < 0.5 mg/kg	65 (27%)
	≥ 0.5 to < 1 mg/kg	37 (15%)
	≥ 1 mg/kg	46 (19%)
Letermovir on day 90	No	191 (79%)
	Yes	52 (21%)
Ever had CMV reactivation before day 90 post‐transplant	No	56 (23%)
	Yes	187 (77%)
Day of 1st CMV reactivation	0–30 days	75 (31%)
	> 30–60 days	97 (40%)
	> 60–90 days	15 (6%)
	No CMV reactivation between day 0–90	56 (23%)
Maximum CMV viral load between day 0–90 post‐transplant	Negative	56 (23%)
	PCR > 0 to < 500 IU/mL or ANT > 0 to < 10 spots	129 (53%)
	PCR ≥ 500 IU/mL or ANT ≥ 10 spots	58 (24%)
Post‐transplant treatment	None	198 (81%)
	Rituximab or Bortezomib	17 (7%)
	Tyrosine kinase inhibitor	9 (4%)
	AML—Azacitidine or FLT3 inhibitors	10 (4%)
	New therapies for GvHD	9 (4%)
Absolute lymphocyte count	Median (Range)	760.0 (70.0–12330.0)
Absolute CD3^+^ cells/μL at day 90	Median (Range)	452.0 (15.4–9728.4)
Absolute CD4^+^ cells/μL at day 90	Median (Range)	212.7 (1.5–1737.7)
Absolute CD8^+^ cells/μL at day 90	Median (Range)	140.9 (1.2–8016.2)

Abbreviations: ALL, acute lymphoblastic leukemia; AML, acute myeloid leukemia; ANT, antigenemia; ATG, anti‐thymocyte globulin; CMV, human cytomegalovirus; CNI, calcineurin inhibitor; GvHD, graft‐versus‐host disease; HLA, human leukocyte antigen; MDS, myelodysplastic syndrome; MMF, mycophenolate mofetil; MTX, methotrexate; PBSC, peripheral blood stem cells; PCR, polymerase chain reaction; PTCy, post‐transplant cyclophosphamide; TCD, T‐cell depletion.

### 3.1 Impact of PT‐Cy, Corticosteroids, and MMF: Timing and Dose Effects 90 Days After HCT

**FIGURE 1:**
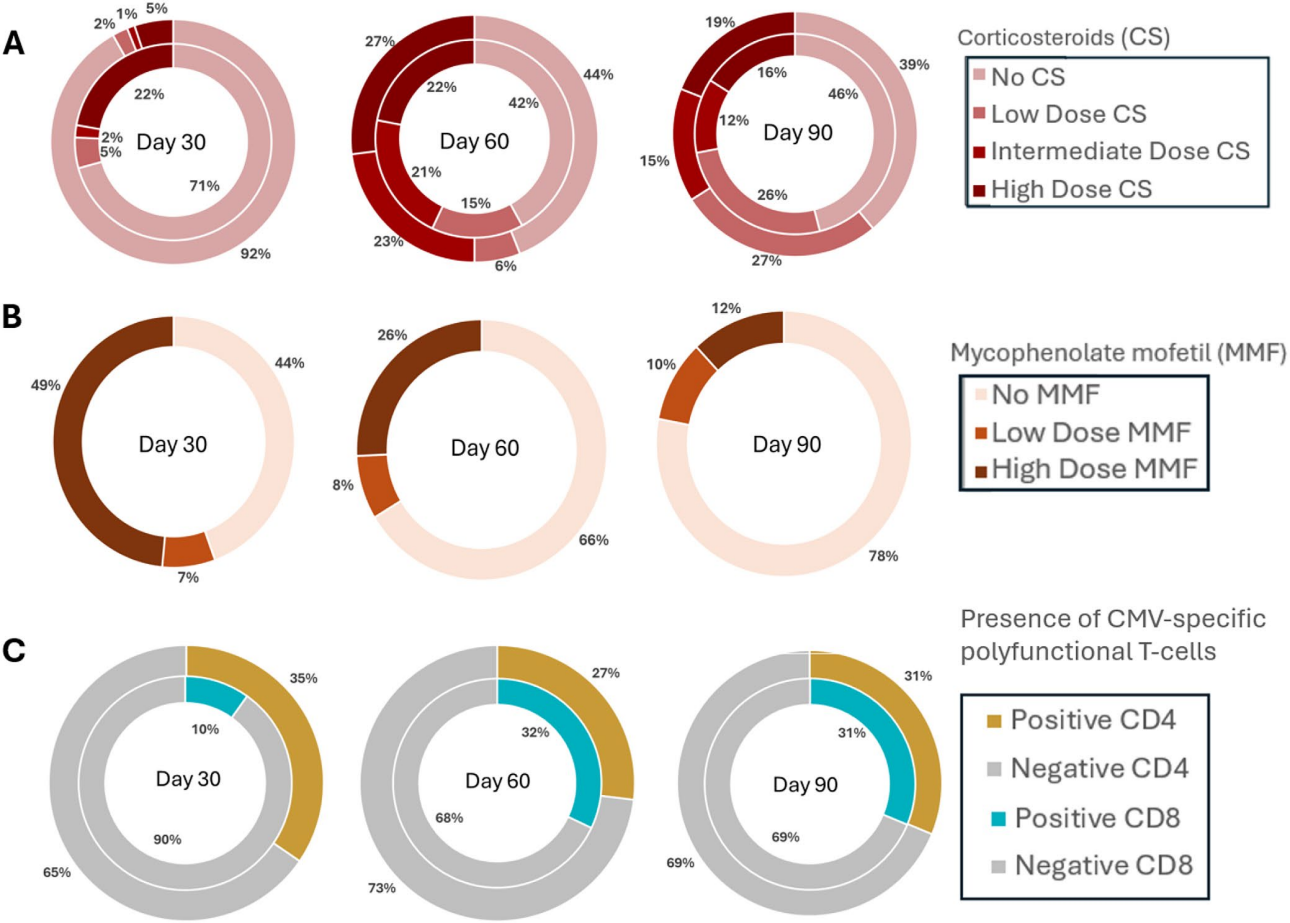
Proportion of patients recieving corticosteroids (CS) by dose, mycophenolate mofetil (MMF) by dose, and developing polyfunctional CMV‐specific T‐cell responses by day 30, 60, and 90 days post‐HCT. (A) The proportion of patients receiving different corticosteroid doses. The inner circle represents the maximum steroid dose administered 0–2 weeks before sample collection, and outer circle maximum steroid dose 2–4 weeks before sample collection—light to dark red, no CS, low dose CS, intermediate dose CS, high dose CS treatment. (B) Proportion of patients according to MMF maximum dose 0–2 weeks before sample collection for each time point—light to dark orange, no MMF, low dose MMF, high dose MMF treatment. (C) Proportion of samples containing > 0.5cell/μL of polyfunctional CMV‐specific T‐cells. The inner circle represents CD8^+^ responses, of which the dark teal is positive, and the gray is negative; the outer circle represents CD4^+^ responses, of which brown is positive and the gray is negative. [Color figure can be viewed at wileyonlinelibrary.com]

Day 90 sample collection indicated that 91 (40%) people received corticosteroid treatment. Low (< 0.5 mg/kg), intermediate (≥ 0.5 to < 1 mg/kg), or high (≥ 1 mg/kg) prednisone‐equivalent doses were used in 51 (21%), 28 (12%) and 19 (8%) patients, respectively. The proportion of different steroid doses within two and four weeks before sample collection is summarized in Figure 1A. Within 14 days before day 90 sample collection, 52 (22%) patients received MMF, with low MMF doses (< 2000 mg) in 24 (10%) and high MMF doses (≥ 2000 mg) in 27 (12%) people (Figure 1B). 33 (14%) individuals received PT‐Cy based GvHD prophylaxis protocols. By day 90, detectable polyfunctional CMV‐specific T‐cell responses (≥ 0.5 cells/μL) were observed in 76 (31%) for each CD4^+^ and CD8^+^ cell subset. (Figure 1C) No differences were observed among PT‐Cy recipients and the rest of the study population (Figure S1). In the subset of patients who underwent IE‐1 testing, we observed lower frequencies of positive responses: only 3 patients (2%) had CD4^+^ T cell counts ≥ 0.5 cells/μL, and a CD8^+^ T cell response was detected in 14 patients (10%).

### 3.2 Enhanced Effects of Corticosteroid Use and MMF

**FIGURE 2:**
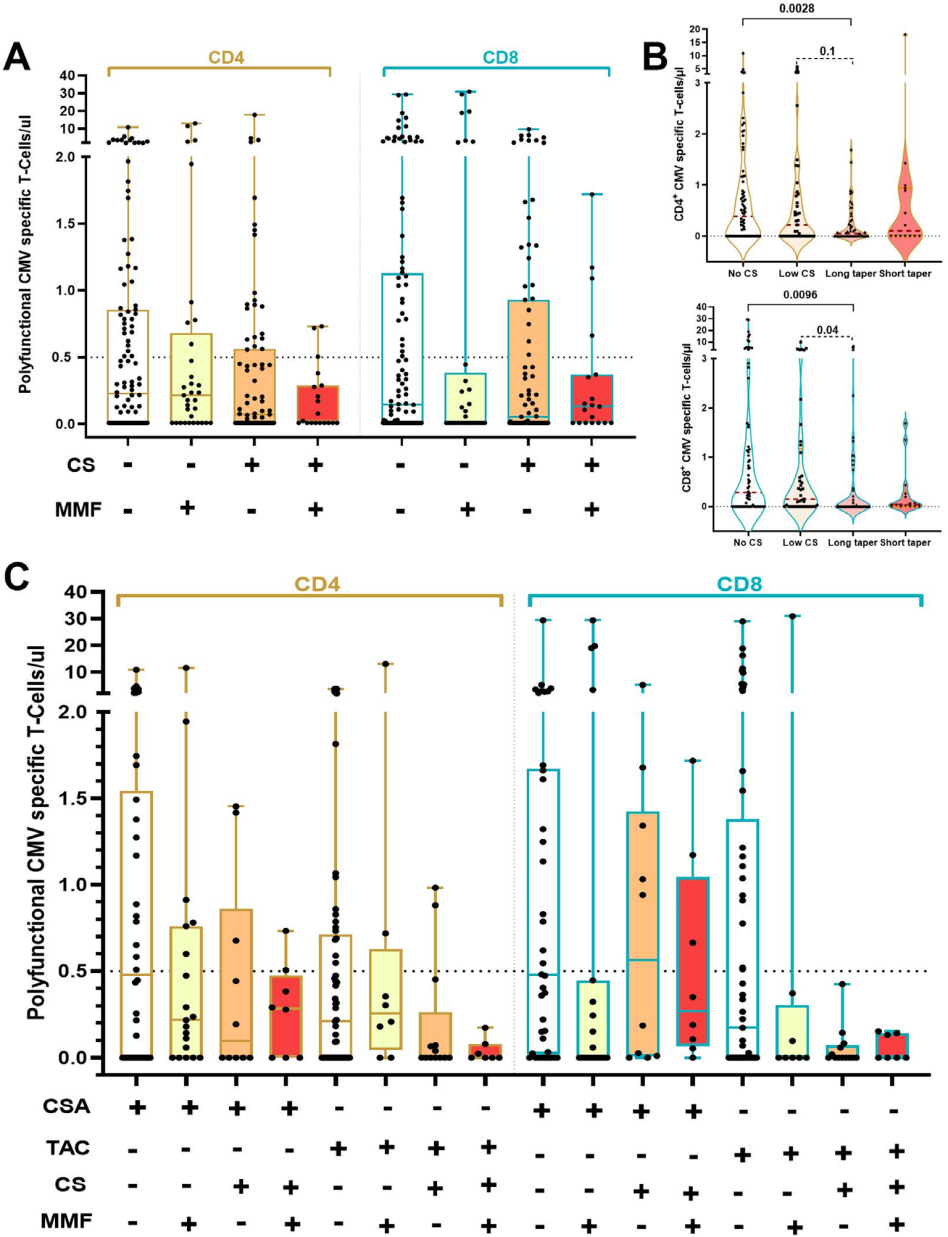
Absolute numbers of CMV specific polyfunctional CD4^+^ (in brown outlines) and CD8^+^ cells (in teal outlines), in different drug categories. (A)—box‐whisker graphs presenting median values with range in patients receiving any dose of corticosteroids (CS) and/or mycophenolate mofetil (MMF), (B) Violin plots showing absolute counts of (C) CD4^+^ and (D) CD8^+^ T‐cells in patients not exposed to prolonged MMF treatment, stratified by steroid dose and timing. and (C) box‐whisker graphs presenting median values with range among patients receiving either cyclosporine (CSA) or tacrolimus (TAC) with additional drugs. *Low CS—dose* < 0.5 mg/kg within 28 days before sampling, Long taper—decrease from high or intermediate dose lasting long than 28 days, Short taper—decrease from high to low CS dose within 28 days. [Color figure can be viewed at wileyonlinelibrary.com]

The concomitant use of MMF and any dose of corticosteroids had a profound suppressive effect on polyfunctional CMV‐specific CD4^+^ and CD8^+^ T cells (Figure 2A), with the suppression being particularly pronounced in the CD4^+^ subset when high‐dose steroids were administered (*p* = 0.04; Figure S2A,B). This additive effect was also observed following SEB stimulation (Figure S2C–F), and similar findings were noted in a subset of after IE‐1 stimulation (Figure S3). However, in the IE‐1 subpopulation, we were unable to perform statistical analyses due to the limited number of events; therefore, these patients were excluded from subsequent parts of the study.

Cytokine production was significantly inhibited by co‐administration of steroids and MMF, with lower frequencies of highly polyfunctional (IFN‐γ^+^ TNFα^+^ IL‐2^+^ 107a^+^ and IFN‐γ^+^ TNFα^+^ IL‐2^+^) CD4^+^ cells (*p* < 0.05; Figure S4A,B).

### 3.3 Effect of Steroid Exposure

To evaluate the impact of steroid exposure on immune recovery, we created an additional model focusing on patients not receiving prolonged MMF treatment (total *n* = 178). These patients were stratified into four groups based on their corticosteroid usage: without corticosteroids (*n* = 74, 41.5%), treated exclusively with low‐dose steroids within 28 days before sampling (*n* = 41, 23%), those who received intermediate or high‐dose steroids for more than 28 days (*n* = 49, 27.5%), and those who underwent a rapid taper from high to low doses within 28 days (*n* = 14, 8.0%). Patients with prolonged intermediate or high‐dose steroid exposure exhibited significantly lower absolute counts of CD4^+^ and CD8^+^ polyfunctional cells (Figure 2B). In contrast, no significant differences were observed in the group that underwent rapid tapering.

### 3.4 Calcineurin Based Immunosuppression in Steroid‐Free MMF Free Population

Next, we analyzed patients who received a CNI‐based GvHD prophylaxis regimen, were not treated with MMF, and did not receive intermediate and high dose corticosteroids at least four weeks prior to sample collection (*n* = 95). By day 90, 38 patients received cyclosporine (CSA) alone and 57 were treated with tacrolimus (TAC).

Polyfunctional CMV‐specific responses were higher in the CSA group, with median CD4^+^ and CD8^+^ T‐cell counts both at 0.47 cells/μL (95% CI: 0–10.96 and 0–29.36, respectively). In contrast, TAC recipients showed lower median counts for both CD4^+^ (0.20 cells/μL, 95% CI: 0–3.7) and CD8^+^ (0.17 cells/μL, 95% CI: 0–28.9) polyfunctional T cells (Figure 2C).

**FIGURE 3:**
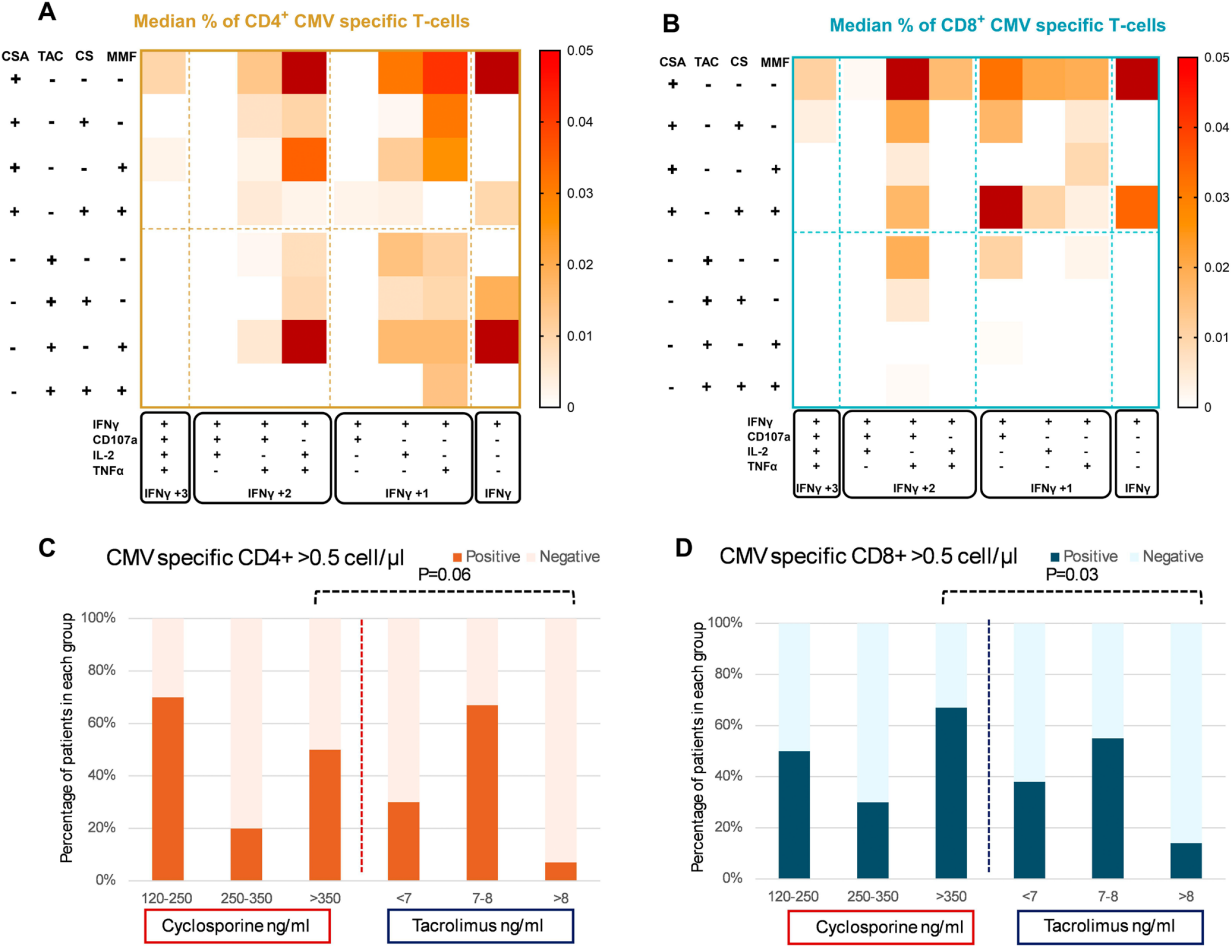
Effects of cyclosporine and tacrolimus on CMV‐specific immunity. Heatmaps showing median percentages of CMV‐specific CD4^+^ (A, outlined in brown) and CD8^+^ (B, outlined in teal) T‐cell subsets producing IFN‐γ in combination with other cytokines, including TNF‐α, IL‐2, and CD107a, according to administered immunosuppressive drugs. Panels A and B include patients receiving corticosteroids cyclosporine (CSA) or tacrolimus (TAC) with or without mycophenolate mofetil (MMF) or high dose corticosteroids (CS). Panels C and D show the frequencies of positive CMV‐specific responses (> 0.5 cells/μL) in groups stratified by different 30‐day average concentrations of cyclosporine or tacrolimus, among CD4^+^ (C) and CD8^+^ (D) T‐cell subsets. [Color figure can be viewed at wileyonlinelibrary.com]

Distinct cytokine production patterns were observed between CSA and TAC recipients. Patients receiving CSA had higher frequencies of highly polyfunctional CMV‐specific T cells, including IFN‐γ^+^ TNFα^+^ IL‐2^+^ 107a^+^ (*p* = 0.086 for CD4^+^, *p* = 0.05 for CD8^+^) and IFN‐γ^+^ TNFα^+^ IL‐2^+^ cells (*p* = 0.003 for CD4^+^), compared to those on TAC (Figures 3A,B, S4C,D).

We performed an additional analysis based on the average concentration of immunosuppressive drugs within the 30 days prior to sampling in 62 patients with available data. In TAC recipients, responses were observed only with lower concentrations (< 8 ng/mL), suggesting a dose‐dependent effect. In contrast, CSA recipients exhibited responses even at drug levels above the reference range. The most pronounced difference between the drugs was observed between patients receiving high doses of CNIs (*p* < 0.05, Figure 3C,D).

### 3.5 Dynamics of Polyfunctional T‐Cell Responses From 30 to 90 Days Post‐HCT

The basic demographics of this subset of patients are summarized in Table S1; data regarding steroid and MMF exposure, CMV reactivation rate and the number of positive CMV specific responses are presented in Figure 1A–C.

### 3.6 Dynamics Among PT‐Cy Recipients

**FIGURE 4:**
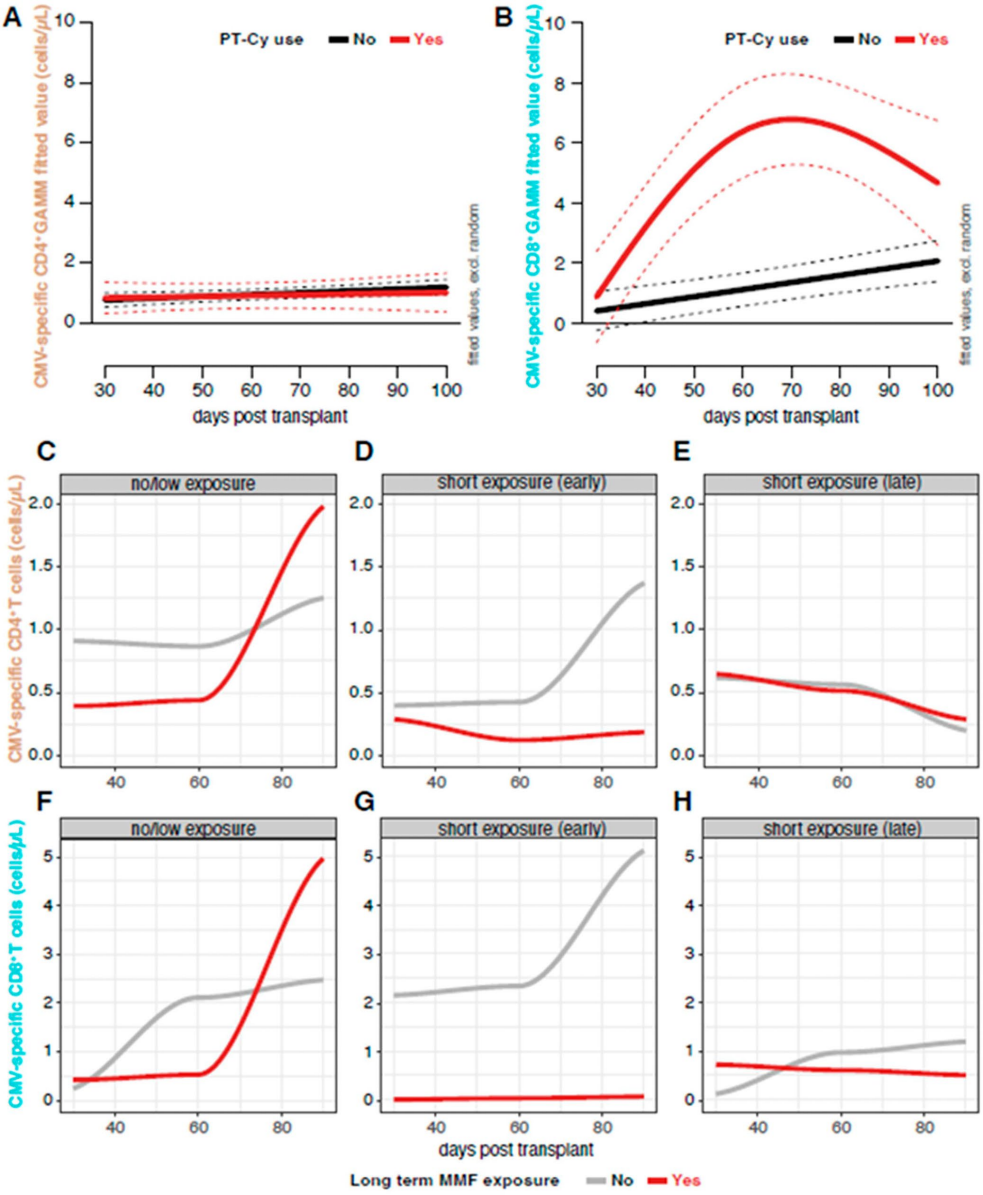
Longitudinal changes in absolute numbers of CMV specific polyfunctional cells in different drug categories. Generalized Additive Model (GAM) showing absolute CMV‐specific CD4+ (A; labeled in brown) and CD8+ (B, labeled in cyan) T‐cell counts over time post‐HCT in PT‐Cy recipients (red line) and patients not receiving PT‐Cy (black line). The model is adjusted for steroid use and participant random effects. Y‐axis: GAMM‐fitted values for CD4 and CD8 cell counts; X‐axis: Days post‐transplant. Smooth function plots (C–H) showing longitudinal changes in CMV‐specific CD4+ and CD8+ T‐cell counts at days 30, 60, and 90 post‐transplant. Panels C, F shows patients without corticosteroid exposure; Panels D, G, early exposure (before day 60); and Panels E, H, late exposure (after day 60). The red line indicates patients with long‐term MMF exposure, gray line indicates patients with no MMF exposure. Lines represent the smoothed conditional means of CD4+ and CD8+ cells across all patients within that group. [Color figure can be viewed at wileyonlinelibrary.com]

To examine the dynamics of immune reconstitution in PT‐Cy recipients, we created additional subset analyses in patients not exposed to intermediate and high dose steroid treatment and not receiving a long course of MMF. Antiviral responses were diminished 30 days after HCT, with comparable results between different groups of GvHD prophylaxis protocols (Figure S5) In GAM models adjusted for steroid exposure, (Figure 4A,B) expansion of polyfunctional CD8+ cells was observed starting 60 days after HCT. None of the patients in this cohort received letermovir prophylaxis. Thus, despite early inhibition of CMV specific responses, rapid reconstitution is observed starting 60 days after HCT.

### 3.7 Effect of Corticosteroids and MMF on Dynamics

When we visualize longitudinal CD4^+^ and CD8^+^ cell counts using smoothed conditional mean plots, the highest number of CMV‐responses were observed in patients not receiving steroid treatment (Figure 4C,F). Treatment with intermediate and high‐dose steroids decreased the observed numbers of polyfunctional CD4^+^ and CD8^+^ cells, and the effect was the strongest with the co‐administration of MMF. We were not able to create similar models for CSA and TAC recipients, due to the limited number of available patient results. Longitudinal changes in three categories stratified according to corticosteroid exposure and MMF use are presented in Figure 4C–H. Overall, this analysis suggests that early and short steroid exposure do not influence immune reconstitution, especially in the absence of MMF.

### 3.8 Impact of Immunosuppressive Drugs on Early Immune Reconstitution After HCT in Univariate and Multivariate Models

**FIGURE 5:**
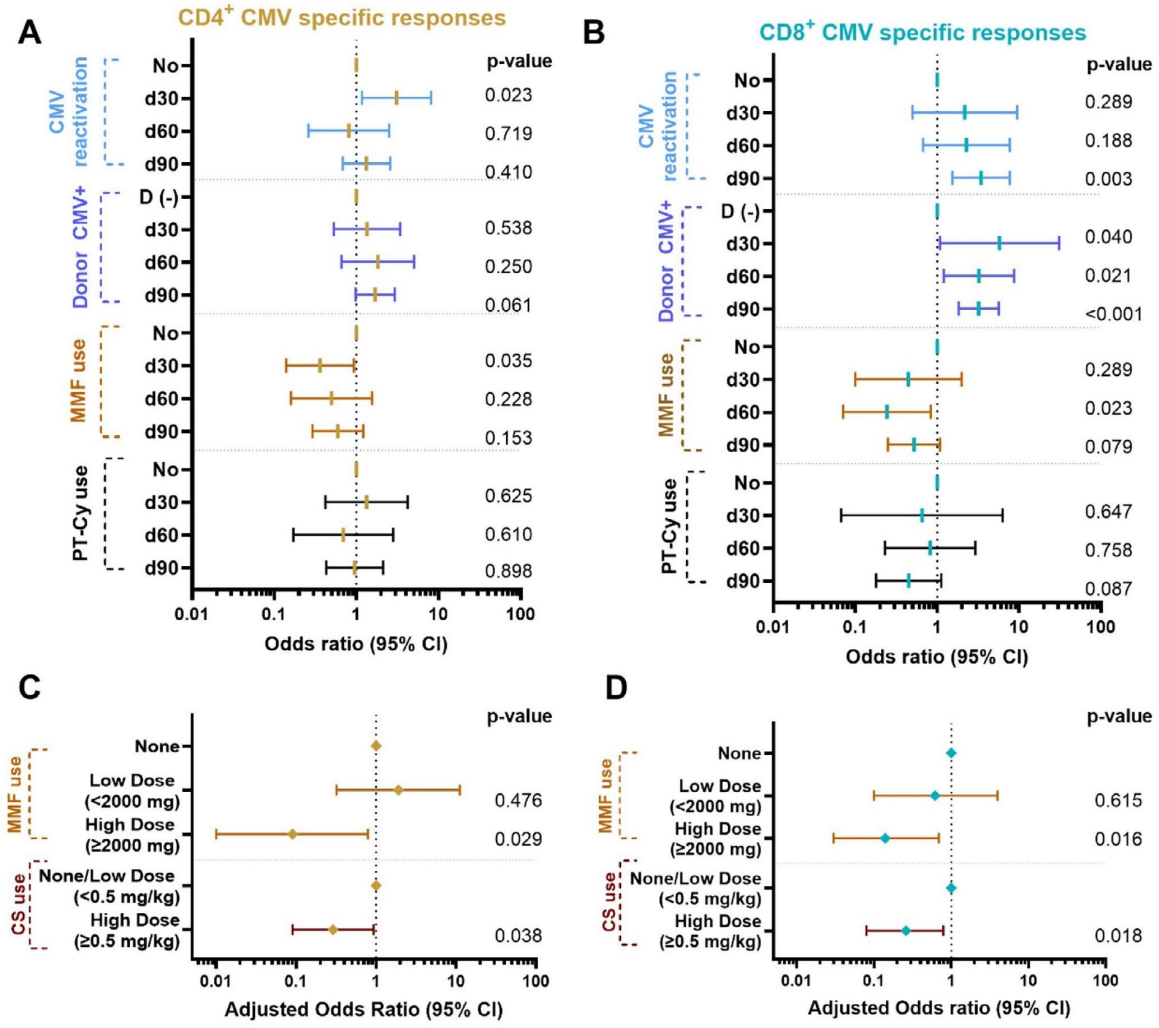
Univariable (UV) and multivariable (MV) logistic regression models for CMV‐specific T‐Cell responses. Panels A and B show UV models including all time points, while panels C and D display MV models at the day 60 post‐transplant time point. Panel A, C: CD4+ T‐cell responses Panel B, D: CD8+ T‐cell responses. Panel C, D: Maximum corticosteroid dose 0–2 weeks prior to sample collection was used. CS, corticosteroids; MMF, mycophenolate mofetil; PT‐Cy, post transplantation. [Color figure can be viewed at wileyonlinelibrary.com]

To determine the impact of different immunosuppressive drugs on early immune CMV‐specific reconstitution, we created univariable models for days 30 and 60 after HCT. In a univariable logistic regression model at day 30, the only factor influencing the odds for lower CD4^+^ responses was treatment with MMF, with a mean difference in polyfunctional CD4^+^ cells of −0.417 cells/μL (95% CI −0.825, −0.010; *p* = 0.04) in a linear model. No effect of PT‐Cy was observed. The response was more robust among those with reactivated CMV infections (CD4^+^) and with seropositive donors (CD8^+^), (Figure 5A,B).

For the day 60 time point, univariable models showed no effect of stem cell source, PT‐Cy use, type of conditioning regimen, or any CMV reactivation on CD4^+^ and CD8^+^ CMV‐specific responses, while donor type was significant for CD8^+^ subset (Figure 5A,B). The only factor affecting both CD4^+^ and CD8^+^ immunity on day 60 was high dose MMF treatment [CD4^+^ OR 0.11 (0.01–0.88), *p* = 0.03, CD8^+^ OR 0.16 (0.03–0.78), *p* = 0.02].

In multivariable models were steroid and MMF doses were categorized separately (low < 2000 mg, high > 2000 mg), a high MMF dose was the strongest factor affecting CD4^+^ and CD8^+^ polyfunctionality by day 60. (Figure 5C,D).

### 3.9 Univariate and Multivariate Modeling of Factors of Immune Reconstitution at Day 90 Post‐HCT

In univariable models, high dose MMF and steroid treatment especially when given 2–4 weeks before sampling, were the most significant factors affecting both CD4^+^ and CD8^+^ responses. Participants receiving PT‐Cy showed a trend towards lower rates of positive CMV specific CD8^+^ responses (OR 0.44 [95% CI 0.18–1.13], *p* = 0.08).

**FIGURE 6:**
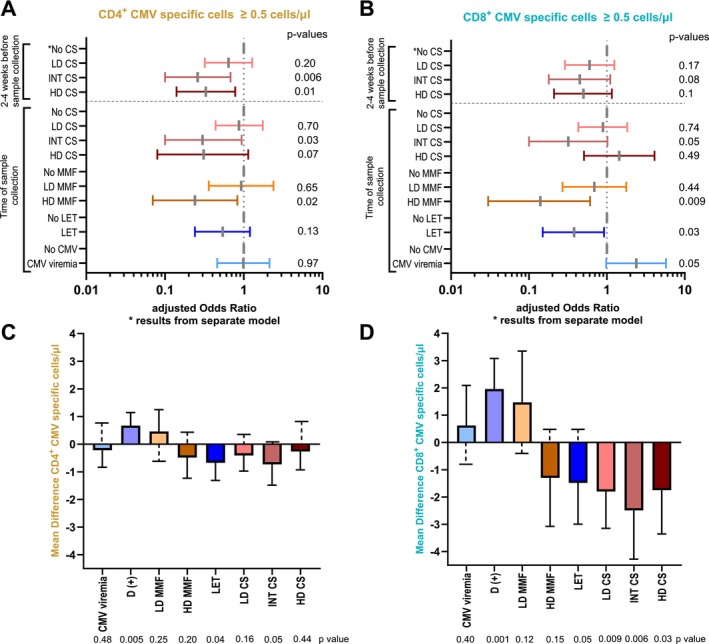
Summary of multivariable regression analyses evaluating factors associated with CMV‐specific T‐cell responses. Panels A and B present results from logistic regression models; Panels C and D show linear regression models. Variables assessed include MMF and corticosteroid dose, letermovir prophylaxis, and CMV reactivation. Data are presented as odds ratios or regression coefficients with 95% confidence intervals. CS, corticosteroids; D (+) – CMV seropositive donor; HD, high dose; INT, intermediate dose; LD, low dose; LET, letermovir; MMF, mycophenolate mofetil. [Color figure can be viewed at wileyonlinelibrary.com]

In multiple multivariate logistic regression models including any CMV viremia, different MMF and steroid doses, and letermovir use, intermediate‐ and high‐dose corticosteroid treatment significantly decreased either CD4^+^ or CD8^+^ responses (Figure 6A,B). Higher levels of CMV viremia did not change the results (data not shown). High doses of MMF (≥ 2000 mg) decreased both CMV‐specific CD4^+^ and CD8^+^ polyfunctional T‐cell responses, though this effect was not significant for doses below 2000 mg. CMV reactivation was a significant factor for increased antiviral response in the CD8^+^ subset, but this response was impaired profoundly by letermovir use (Figure 6B). In additional model, positive donor CMV serostatus increased the odds to achieve both CD4^+^ (aOR 1.64, 95% CI 0.93–2.91, *p* = 0.09) and CD8^+^ (aOR 3.28, 95% CI 1.78–6.04, *p* < 0.001) responses by day 90 post‐HCT and higher absolute numbers of polyfunctional cells were observed in patients transplanted from CMV‐seropositive donors (Figure S6).

There was greater impairment of responses when steroid treatment (≥ 0.5 mg/kg) was administered within 2–4 weeks prior to sample collection. The mean difference was statistically significant for both CD4^+^ cells (OR −0.81 cells/μl [95% CI −1.51, −0.1]) and CD8^+^ cells (OR −2.33 cells/μl [95% CI −4.03, −0.64]). Multivariable linear regression models showed similar results for the association with T cell response magnitudes (Figure 6C,D).

## 4 Discussion

In this study, we conducted deep dive analyses to identify factors influencing CMV‐specific polyfunctional T‐cell responses, with particular emphasis on the dose and timing of commonly used immunosuppressive agents, including PT‐Cy, MMF, CSA, TAC, and corticosteroids. Our findings reveal that different immunosuppressive drugs exert distinct effects on immune recovery. Notably, prolonged exposure to intermediate or high‐dose corticosteroids severely impaired CMV‐specific CD4^+^ and CD8^+^ T‐cell function, while a rapid tapering strategy appeared to mitigate this impact and support immune reconstitution. High doses of MMF, emerged as the most potent suppressor of CMV‐specific immunity, significantly reducing both proliferative capacity and cytokine production. Importantly, although CSA and TAC are both calcineurin inhibitors, they differ in their immunomodulatory profiles: TAC was associated with lower IL‐2 and TNFα production and reduced absolute numbers of CMV‐specific T cells compared to CSA. These findings underscore the potential to tailor immunosuppressive regimens to preserve antiviral immunity, particularly in patients with refractory or resistant CMV infection or those being considered for virus‐specific T‐cell therapies.

PT‐Cy has been associated with an early reduction of T‐cell receptor diversity after HCT [33]. The immunologic bottleneck likely contributes to the increased incidence of viral complications among recipients of this GvHD prophylaxis protocol [9], especially in early post HCT period [8]. Both PT‐Cy and MMF have been independently associated with an increased risk of early CMV reactivation, particularly at higher viral load thresholds [8]. However, our study suggests that this early susceptibility may be mitigated by a rapid reconstitution of CMV‐specific T‐cell responses observed around day 60 post‐transplant. This early immune recovery may contribute to enhanced long‐term viral control in recipients of PT‐Cy‐based GvHD prophylaxis [8]. The effect of PT‐Cy on CMV specific immunity is complex and can be influenced by co‐administrated drugs and the occurrence of GvHD.

Currently used PT‐Cy based protocols include high MMF doses administered within the first month post‐HCT [34, 35], however, with growing numbers of mismatched donor HCT [36], and the increasing trend towards limiting PT‐Cy dosing [37, 38, 39], the routine dosage and timing of MMF may vary across HCT recipients in the future. The combination of CNI‐MMF with PT‐Cy, improves GvHD‐free, relapse‐free survival compared to standard CNI‐methotrexate regimens [40] and has become the standard of care, even beyond the haploidentical transplant setting [34, 41, 42]. However, recent studies have shown that CNI‐MMF based prophylaxis is associated with a higher risk of CMV reactivation and increased mortality in CMV seropositive HCT recipients [8, 43]. Mycophenolate inhibits cell proliferation and affects mostly B and T lymphocytes [44]. Cell cytotoxicity seems to be unaffected during short‐term MMF exposure, however, long term MMF treatment influences cell proliferation, viability, and cytotoxicity [45]. In recently conducted or ongoing VST clinical trials, MMF administration is not routinely considered as a potential factor influencing antiviral responses. However, we observed that in vivo CMV‐specific responses were consistently significantly diminished at 30, 60, and 90 days after HCT in patients receiving high‐dose MMF treatment, suggesting that reduction of MMF should be considered if a high dose is used.

While the impact of PT‐Cy on viral infections and immune reconstitution has been the focus of recent research [9] recent data from SOT suggest that the CNIs affect viral immunity and reactivation differently [18]. Indeed, studies involving SOT recipients diagnosed with BK and respiratory viruses showed that calcineurin inhibitors shape different antiviral responses, providing more protection in CSA recipients [15, 18, 46, 47]. Higher tacrolimus concentrations inhibit BK virus specific responses in over 50% of patients, suggesting a dose‐dependent immunosuppressive effect on antiviral T‐cell function [48]. In contrast, cyclosporine appears to exert a distinct in vitro influence on BK, effectively suppressing the initial peak and reducing the likelihood of subsequent viral reactivation [49]. Older studies showed in vitro inhibition of herpes simplex virus production by cyclosporine [50], however its effects on other herpesviruses, including CMV and CMV‐specific immunity, remain unexplored.

That the choice of CNIs may also affect CMV‐specific T cell immunity in HCT recipients is a novel finding. Our study demonstrated reduced CMV‐specific responses in TAC recipients, particularly at higher therapeutic doses, whereas CSA levels did not show a significant threshold effect. Altogether, concomitant use of tacrolimus with MMF may inhibit both proliferative and secretory functions and contribute to the lower antiviral responses and higher incidence of CMV reactivation in this group of HCT recipients. Whether there is virologic correlate to these findings will require additional studies. Modulation of immunosuppressive treatment by decreasing the immunosuppressant doses or switching to mTOR inhibitors or CSA may be beneficial for patients suffering from refractory or resistant CMV infection qualified for virus specific therapies, as was demonstrated in SOT recipients [51, 52, 53, 54, 55]. We were not able to create similar models for sirolimus with this dataset due to limited number of events, but the beneficial effects of mTOR inhibitors in controlling CMV infection in both SOT and HCT recipients are well established [51, 55, 56].

Finally, it is well recognized that CMV‐specific responses are inhibited by very high dose steroid treatment [11] but the data originates from historical populations where both transplantation techniques and older, culture‐based CMV‐specific immunity assessments differed significantly compared to the current era. Our findings indicate that both the timing and dosage of corticosteroid critically influence CMV‐specific immune responses. Moreover, high‐dose steroid administration two to four weeks prior to sampling exerted a more substantial immunosuppressive effect compared to equivalent doses given shortly before or at the time of sampling, likely reflecting the cumulative impact of prolonged steroid treatment. Importantly, we confirmed that patients who underwent a rapid taper from intermediate or high‐dose steroids were able to mount CMV‐specific immune responses, suggesting that timely reduction in steroid exposure may allow for partial restoration of T‐cell functionality. This observation supports steroid dose manipulation in patients with complicated CMV infections or prior to planned VST to optimize treatment outcomes by reduction of all significant drugs affecting CMV specific polyfunctionality. Optimizing the dose and timing of immunosuppressive drugs could help include more patients in future clinical trials of virus‐specific T‐cell therapies, making these treatments available to more patients with recurrent infections.

In conclusion, we identified several modifiable factors that may optimize polyfunctional CMV‐specific T‐cell responses among seropositive HCT recipients. Long exposure to intermediate to high doses of steroids, along with concurrent high‐dose MMF treatment, significantly suppressed both CD4^+^ and CD8^+^ polyfunctional CMV responses. Notably, the strength of T cell responses varied between patients receiving cyclosporine and those on TAC, with the TAC group exhibiting reduced cytokine production and lower absolute numbers of polyfunctional T cells. Conversely, PT‐Cy did not appear to impair immune reconstitution, which remained robust and comparable to other immunosuppressive regimens. These novel insights indicate a paradigm shift and highlight the critical need to refine immunosuppressive strategies in the management of viral infections, offering valuable guidance for the design of future T‐cell therapy trials and presenting new opportunities to improve the management of refractory or resistant CMV infection in everyday clinical practice. Prospective interventional studies are needed to assess the efficacy of these new management strategies.

## Author Contributions

A.S.‐K. and M.B. contributed to conception and design; A.S.‐K., F.Y.L., D.Z., T.S.‐A., B.C.E., S.D.R. and M.M. were responsible for the provision of study materials and data collection; A.S.‐K., M.B., H.X., W.L., F.Y.L. conducted data analysis and interpretation; A.S.‐K. and M.B. prepared the initial draft of the manuscript; all authors provided critical review and final approval of the manuscript and are accountable for all aspects of the work.

## Funding

A.S.K. received support from the Medical University of Gdansk and the Joel D. Meyers Scholarship Endowment, Fred Hutchinson Cancer Center, Seattle, WA. National Institutes of Health (CA15704 [H.X., W.L.], K23AI163343 [D.Z.]). Fred Hutchinson Cancer Research Center, Vaccine and Infectious Disease Division (Sample Repository).

## Ethics Statement

The study was approved by the Fred Hutch Institutional Review Board, and the patients provided written informed consent before sample collection.

## Conflicts of Interest

M.B. reports research support from Merck, Moderna and Oxford Immunotec (Research supplies), and consulting fees from Merck, AlloVir, Symbio, Takeda, Evrys Bio, and Moderna. The remaining authors declare no conflicts of interest.

## Supporting information


**Table S1:** Selected Ongoing or Completed Clinical Trials of Virus‐Specific T‐Cell Therapies and Key Immunosuppression‐Related Exclusion Criteria.
**Table S2:** Basic demographics of the study group—subset of patients with samples available for day 30 and 60 post HCT analyses.
**Figure S1:** Box‐whisker graphs presenting median values with range after pp65 stimulation at day 90 after transplantation in patients according to whether they received post‐transplant cyclophosphamide (PT‐Cy). Absolute numbers of polyfunctional CD4^+^ (in brown outlines) and CD8^+^ cells (in teal outlines). Heatmap presenting 25th, 50th and 75th percentile for each category.
**Figure S2:** Absolute numbers of polyfunctional CD4^+^ (in brown outlines) and CD8^+^ cells (in teal outlines), in different drug categories. (A, B)—box‐whisker graphs presenting median values with range after pp65 stimulation in patients receiving high dose (HD > 0.5 m/kg) of steroids (CS) and/or mycophenolate mofetil (MMF), (C‐F) box‐whisker graphs presenting median values with range after SEB stimulation in patients receiving any dose (C, D) or high dose (E, F) of corticosteroids and/or MMF.
**Figure S3:** Frequencies of Polyfunctional (IFN‐γ + ≥ 1) and Monofunctional (IFN‐γ) CMV‐Specific CD4⁺ (A, C) and CD8⁺(B, D) T Cells by Cytokine Profile and Immunosuppressive Regimen (A, B: Corticosteroids, MMF; C, D: Cyclosporine (CSA)), Tacrolimus (TAC) among patients not exposed to MMF or steroids within 28 before sample collection Distinct cytokine production patterns were observed between CSA and TAC recipients. Results with statistical significance are marked with *. Patients receiving CSA had higher frequencies of highly polyfunctional CMV‐specific T cells, including IFN‐γ^+^ TNFα^+^ IL‐2^+^ 107a^+^ (*p* = 0.086 for CD4⁺, *p* = 0.05 for CD8⁺) and IFN‐γ^+^ TNFα^+^ IL‐2^+^ cells (*p* = 0.003 for CD4⁺), compared to those on TAC. Among CD8⁺ cells, the CSA group also showed a significantly higher proportion of IFN‐γ^+^ IL‐2⁺ producers (*p* = 0.01) and a trend towards increased IFN‐γ⁺ TNFα⁺ cells (*p* = 0.06). Additionally, both CD4⁺ and CD8⁺ subsets in the CSA group exhibited higher frequencies of cells producing IFN‐γ alone (*p* = 0.002 for CD4⁺, *p* = 0.09 for CD8⁺).
**Figure S4:** Dynamics of CMV‐specific immune reconstitution in patients not exposed to MMF and steroids at the time of sample collection stratified by PT‐Cy administration, panel A—CD4^+^, panel B—CD8^+^.

## Data Availability

The data that support the findings of this study are available from the corresponding author, (M.B), upon reasonable request and after execution of a data transfer agreement.
