# Supplementary material for: New Insights Into Factors Shaping CMV‐Specific T‐Cell Polyfunctionality After Hematopoietic Cell Transplantation
Source: Am J Hematol. 2025 Dec 1;101(2):255–68. doi: 10.1002/ajh.70152 (PMC12766359; doi:10.1002/ajh.70152)
Supplement: Supplementary file 1 — Table S1: Selected Ongoing or Completed Clinical Trials of Virus‐Specific T‐Cell Therapies and Key Immunosuppression‐Related Exclusion Criteria. Table S2: Basic demographics of the study group—subset of patients with samples available for day 30 and 60 post HCT analyses. Figure S1: Box‐whisker graphs presenting median values with range after pp65 stimulation at day 90 after transplantation in patients according to whether they received post‐transplant cyclophosphamide (PT‐Cy). Absolute numbers of polyfunctional CD4+ (in brown outlines) and CD8+ cells (in teal outlines). Heatmap presenting 25th, 50th and 75th percentile for each category. Figure S2: Absolute numbers of polyfunctional CD4+ (in brown outlines) and CD8+ cells (in teal outlines), in different drug categories. (A, B)—box‐whisker graphs presenting median values with range after pp65 stimulation in patients receiving high dose (HD > 0.5 m/kg) of steroids (CS) and/or mycophenolate mofetil (MMF), (C‐F) box‐whisker graphs presenting median values with range after SEB stimulation in patients receiving any dose (C, D) or high dose (E, F) of corticosteroids and/or MMF. Figure S3: Frequencies of Polyfunctional (IFN‐γ + ≥ 1) and Monofunctional (IFN‐γ) CMV‐Specific CD4⁺ (A, C) and CD8⁺(B, D) T Cells by Cytokine Profile and Immunosuppressive Regimen (A, B: Corticosteroids, MMF; C, D: Cyclosporine (CSA)), Tacrolimus (TAC) among patients not exposed to MMF or steroids within 28 before sample collection Distinct cytokine production patterns were observed between CSA and TAC recipients. Results with statistical significance are marked with *. Patients receiving CSA had higher frequencies of highly polyfunctional CMV‐specific T cells, including IFN‐γ+ TNFα+ IL‐2+ 107a+ (p = 0.086 for CD4⁺, p = 0.05 for CD8⁺) and IFN‐γ+ TNFα+ IL‐2+ cells (p = 0.003 for CD4⁺), compared to those on TAC. Among CD8⁺ cells, the CSA group also showed a significantly higher proportion of IFN‐γ+ IL‐2⁺ producers (p = 0.01) and a trend towards incre [file AJH-101-255-s001.docx]

Supplementary data:

**Flow cytometry methodology**

All reagents were purchased from BD Biosciences, San Jose, CA, unless otherwise specified. Cryopreserved PBMC were thawed in enriched media (RPMI-HEPES; Gibco, Gaithersburg, MD) with 10% human AB serum (Sigma-Aldrich), 2 mM L-glutamine (Gibco) and 1% antibiotic/antimycotic (Sigma-Aldrich). PBMCs were incubated with anti-CD107a PECy5 antibody for ten minutes, followed by the addition of the co-stimulatory antibodies anti-CD28 (1 mg/mL) and anti-CD49d (1 mg/mL). Cells were then stimulated with pp65 pepmix (2 mg/ml; JPT Peptide Technologies, Berlin, Germany), *Staphylococcus aureus* enterotoxin B (0.05 mg/mL; Sigma-Aldrich) or 0.5% DMSO for six hours at 37°C and 5% CO_2_. Brefeldin A (10 mg/mL, Sigma-Aldrich)/Golgi stop was added during the last four hours of incubation. Samples were held overnight at 4^0^C and then stained for flow cytometric analysis the following day.

After incubation with EDTA (20 mM), samples were incubated first with fixable viability dye (Invitrogen/Thermo Fisher, Waltham, MA) for 30 minutes in 1X PBS, washed, incubated successively with 1X FACSlyse and 1X FACSperm or in one step with Cytofix/Cytoperm, washed with FACS wash buffer (0.5% bovine serum albumin in 1X PBS), and then incubated for 30 minutes in the dark at room temperature with a cocktail of fluorescently labelled antibodies against CD3, CD4, CD8, IFNg APC, IL-2 and TNFa. Finally, samples were fixed with 1% paraformaldehyde (Sigma-Aldrich) and acquired (100,000 – 400,000 events) on an LSR-II flow cytometer (BD Biosciences) within 24 hours of staining. All antibodies were titrated for optimum performance, and appropriate single-color compensation and fluorescence minus-one controls were run. Data were analyzed with FlowJo software, version 9.9.6.

**Supplemental Table1.** Selected Ongoing or Completed Clinical Trials of Virus-Specific T-Cell Therapies and Key Immunosuppression-Related Exclusion Criteria

| **Clinical trial** | Clinical Trials ID | Major Exclusions related to IST |
| --- | --- | --- |
| **Treatment of Cytomegalovirus (CMV) Infections With Viral-Specific T Cells** | NCT03798301 | Patient receiving steroids (>1.0 mg/kg body weight (BW) prednisone equivalent) at the time of T-cell transfer;  Patient treated with Thymoglobulin (ATG), Alemtuzumab or T-cell immunosuppressive monoclonal antibodies within 28 days. |
| **Donor Cytomegalovirus-Specific Cytotoxic T-Lymphocytes in Treating Patients With a Persistent Cytomegalovirus Infection** | NCT02210078 | Patients receiving prednisone >0.5 mg/kg/day at time of enrollment, or have received ATG, donor lymphocyte infusion (DLI) or Campath within 28 days of enrollment |
| **Prophylaxis of Cytomegalovirus Infection With Adoptive Cell Inmunotherapy (INMUNOCELL)** | NCT04056533 | Patients receiving corticosteroid (dose of 0.5mg/kg/day of prednisone or equivalent) at infusion.  Patients who received ATG, donor lymphocytes or alemtuzuamb, 28 days pre-infusion. |
| **Trial of Third Party Donor Derived CMVpp65 Specific T-cells for The Treatment of CMV Infection or Persistent CMV Viremia After Allogeneic Hematopoietic Stem Cell Transplantation** | NCT02136797 | Patients requiring high doses of glucocorticosteroids (≥ 0.3 mg/kg prednisone or its equivalent) |
| **CMV Specific T Cell Therapy After Allogeneic Stem Cell Transplantation.** | NCT03067155 | Patient on corticosteroids > 0.5mg/kg. Patient can still be on therapeutic doses of immunosuppressive therapy, but these will be tapered to the lowest possible dose, as is part of standard care in case of CMV reactivation.  Patient that has received ATG < 1 month prior to infusion or Campath < 1 year prior to infusion |
| **R-MVST Cells for Treatment of Viral Infections in Children and Young Adults** | NCT06926894 | Patients who receive corticosteroids at ≥ 0.5mg/kg prednisone or equivalent  Patients who received anti-thymocyte globulin (ATG, Alemtuzumab (Campath), or other T-Cell immunosuppressive monoclonal antibodies in the last 28 days. Patients who received methotrexate, or other antimetabolite-type immunosuppressants that are toxic to proliferating T cells in the last 7 days  Patients who received checkpoint inhibitor agents (e.g., nivolumab, pembrolizumab, ipilimumab) within 3 drug half-lives of the most recent dose to the infusion of R-MVST. |
| **Multivirus-specific T Cells in the Treatment of Refractory CMV and/​or EBV Infection After Allo-HSCT** | NCT06075927 | Received DLI, other CTL, CAR-T, NK and other cell therapies, T cell monoclonal antibody immunosuppressants, or participated in any other clinical research related to drugs and medical devices within 28 days before enrollment.  Prednisone or its equivalent hormone is greater than 0.5 mg/kg/ day when enrolled. |
| **T Lymphocytes for the Treatment of AdV, CMV, EBV, BKV and Aspergillus Fumigatus Infections After Allogeneic Stem Cell Transplantation (Penta-STs-001)** | NCT05471661 | Received ATG, or Campath or other T cell immunosuppressive monoclonal antibodies in the last 28 days.  Steroids > 0.5 mg/kg/day prednisone. |
| **R-MVST Cells for Treatment of Viral Infections** | NCT05183490 | Patients who receive corticosteroids at ≥ 0.5mg/kg prednisone or equivalent.  Patients who received anti-thymocyte globulin (ATG, Alemtuzumab (Campath), or other T-Cell immunosupressive monoclonal antibodies in the last 28 days.  Patients who received methotrexate, or other antimetabolite-type immunosuppressants that are toxic to proliferating T cells in the last 7 days.  Patients who received checkpoint inhibitor agents (e.g., nivolumab, pembrolizumab, ipilimumab) within 3 drug half-lives of the most recent dose to the infusion of R-MVST |
| **Adoptive T-cell Therapy for Resistant Viral Infections After Allogeneic HSCT (VS-TC)** | NCT05075837 | CS not included into exclusion criteria |
| **Multivirus-specific T-cell Transfer Post SCT vs AdV, CMV and EBV Infections (TRACE)** | NCT04832607 | Patient receiving steroids (>1 mg/kg BW Prednisone equivalent) at Screening. |
| **Virus-specific Activated T Lymphocytes From a Donor in Hematopoietic Progenitor Transplanted Patients** | NCT04018261 | Corticosteroid ≥ 0.5mg/kg regardless the indication |
| **TETRAVI Multivirus CTL for Treatment of EBV, CMV, Adenovirus, and BK Infections Post Allogeneic SCT. (TETRAVI)** | NCT04013802 | Patients receiving ATG, Campath or other immunosuppressive T cell monoclonal antibodies within 28 days of screening for enrollment.  Clinical status at enrollment to allow tapering of steroids to equal or less than 0.5 mg/kg/day methylprednisolone (or equivalent). |
| **Treatment of Cytomegalovirus (CMV) Infections With Viral-Specific T Cells** | NCT03798301 | Patient receiving steroids (>1.0 mg/kg body weight (BW) prednisone equivalent) at the time of T-cell transfer.  Patient treated with Thymoglobulin (ATG), Alemtuzumab or T-cell immunosuppressive monoclonal antibodies within 28 days. |
| **Virus Specific Cytotoxic T-Lymphocytes (CTLs) for Refractory Cytomegalovirus (CMV)** | NCT03266640 | Patient receiving steroids (>0.5 mg/kg prednisone equivalent) at the time of CMV CTL infusion  Thymoglobulin (ATG), Alemtuzumab or T cell immunosuppressive monoclonal antibodies within 30 days |

**Supplemental Table 2.** Basic demographics of the study group – subset of patients with samples available for day 30 and 60 post HCT analyses.

| **Variables** | **Categories** | **Day 30 (N=81)** | **Day 60 (N=78)** |
| --- | --- | --- | --- |
| Age | Median (Range) | 55.9 (22.6-75.7) | 55.7 (22.6-75.7) |
| Sex | Female | 27 (33%) | 25 (32%) |
|  | Male | 54 (67%) | 53 (68%) |
| Donor sex | Female | 31 (38%) | 29 (37%) |
|  | Male | 50 (62%) | 49 (63%) |
| Ethnicity | Hispanic or Latino | 3 (4%) | 3 (4%) |
|  | Not Hispanic or Latino | 78 (96%) | 75 (96%) |
| Race | American Indian or Alaska Native | 1 (1%) | 1 (1%) |
|  | Asian | 3 (4%) | 3 (4%) |
|  | Multiple | 1 (1%) | 1 (1%) |
|  | Native Hawaiian or other Pacific | 1 (1%) | 1 (1%) |
|  | White | 75 (93%) | 72 (92%) |
| Cell source | Peripheral stem cells | 73 (90%) | 71 (91%) |
|  | Bone Marrow | 8 (10%) | 7 (9%) |
| Donor Type | Related matched | 26 (32%) | 25 (32%) |
|  | Mismatched related | 11 (14%) | 11 (14%) |
|  | Unrelated matched | 38 (47%) | 36 (46%) |
|  | Unrelated mismatched | 6 (7%) | 6 (8%) |
| Underlying disease | AML/MDS | 34 (42%) | 31 (40%) |
|  | ALL | 4 (5%) | 4 (5%) |
|  | Lymphoproliferative | 34 (42%) | 34 (44%) |
|  | Chronic myeloid | 9 (11%) | 9 (12%) |
| PT-Cy | No | 66 (81%) | 64 (82%) |
|  | Yes | 15 (19%) | 14 (18%) |
| TCD or ATG or Campath | No | 79 (98%) | 76 (97%) |
|  | Yes | 2 (2%) | 2 (3%) |
| GVHD prophylaxis | CNI+MMF | 31 (38%) | 29 (37%) |
|  | CNI+MTX | 26 (32%) | 26 (33%) |
|  | PT-Cy | 15 (19%) | 14 (18%) |
|  | Sirolimus based | 7 (9%) | 7 (9%) |
|  | Other | 2 (2%) | 2 (3%) |
| Conditioning regimen | Myeloablative | 35 (43%) | 34 (44%) |
|  | Non-myeloablative | 46 (57%) | 44 (56%) |
| CMV specific CD4+ positive response | No | 53 (65%) | 57 (73%) |
|  | Yes | 28 (35%) | 21 (27%) |
| CMV specific CD8+ positive response | No | 73 (90%) | 53 (68%) |
|  | Yes | 8 (10%) | 25 (32%) |
| Donor CMV serostatus | - | 50 (62%) | 49 (63%) |
|  | + | 31 (38%) | 29 (37%) |
| Acute GVHD | Grade 0-2 | 74 (91%) | 71 (91%) |
|  | Grade 3-4 | 7 (9%) | 7 (9%) |
| Chronic GVHD | No | 30 (37%) | 29 (37%) |
|  | Yes | 51 (63%) | 49 (63%) |
| Post HCT treatment | None | 66 (81%) | 58 (74%) |
|  | Rituximab or Bortezomib | 13 (16%) | 14 (18%) |
|  | Tyrosine kinase inhibitors | 1 (1%) | 3 (4%) |
|  | Azacitidine or FLT inhibitors | 1 (1%) | 2 (3%) |
| CMV reactivation before sample collection | No | 54 (67%) | 20 (26%) |
|  | Yes | 27 (33%) | 58 (74%) |
| Maximum CMV viral load before sample collection | Negative | 54 (67%) | 20 (26%) |
|  | PCR >0 - <500 IU/mL or ANT >0 - <10 spots | 22 (27%) | 39 (50%) |
|  | PCR ≥500 IU/mL or ANT ≥10 spots | 5 (6%) | 19 (24%) |
| Steroid dosage at the time of sample collection | None | 57 (70%) | 37 (47%) |
|  | >0 - <0.5 mg/kg | 5 (6%) | 18 (23%) |
|  | ≥0.5 - <1 mg/kg | 8 (10%) | 14 (18%) |
|  | ≥1 mg/kg | 11 (14%) | 9 (12%) |
| Maximum steroid within 0-2 weeks before sample collection | None | 57 (70%) | 33 (42%) |
|  | >0 - <0.5 mg/kg | 4 (5%) | 12 (15%) |
|  | ≥0.5 - <1 mg/kg | 2 (2%) | 16 (21%) |
|  | ≥1 mg/kg | 18 (22%) | 17 (22%) |
| Maximum steroid within 2-4 weeks before sample collection | None | 73 (90%) | 34 (44%) |
|  | >0 - <0.5 mg/kg | 2 (2%) | 5 (6%) |
|  | ≥0.5 - <1 mg/kg | 1 (1%) | 18 (23%) |
|  | ≥1 mg/kg | 5 (6%) | 21 (27%) |
| MMF 0-14 days prior to sample collection | No | 36 (44%) | 51 (65%) |
|  | Yes | 45 (56%) | 27 (35%) |
| MMF exposure days | No MMF | 36 (44%) | 35 (45%) |
|  | 0-35 days | 44 (54%) | 15 (19%) |
|  | >35 days | 1 (1%) | 28 (36%) |
| MMF dosage | None | 36 (44%) | 52 (67%) |
|  | Low (<2000) | 6 (7%) | 6 (8%) |
|  | High (>=2000) | 39 (48%) | 20 (26%) |
| Absolute lymphocyte count | Median (Range) | 610.0 (20.0-7020.0) | 825.0 (0.0-3960.0) |
| abs CD3+ cells/ul | Median (Range) | 314.1 (1.4-4190.9) | 493.7 (0.0-2362.6) |
| abs CD4+ cells/ul | Median (Range) | 162.7 (0.9-1311.8) | 212.2 (0.0-940.4) |
| abs CD8+ cells/ul | Median (Range) | 94.5 (0.2-2414.0) | 145.9 (0.0-1507.3) |

Abbreviations: AML – acute myeloid leukemia, MDS – myelodysplastic syndrome, ALL – acute lymphoblastic leukemia, CMV – human cytomegalovirus, PBSC – peripheral blood stem cells, HLA – human leukocyte antigen, TCD – T-cell depletion, ATG – anti-thymocyte globulin, GvHD – graft-versus-host disease, CNI – calcineurin inhibitor, MMF – mycophenolate mofetil, MTX - methotrexate, PTCy – post-transplant cyclophosphamide, PCR – polymerase chain reaction, ANT - antigenemia


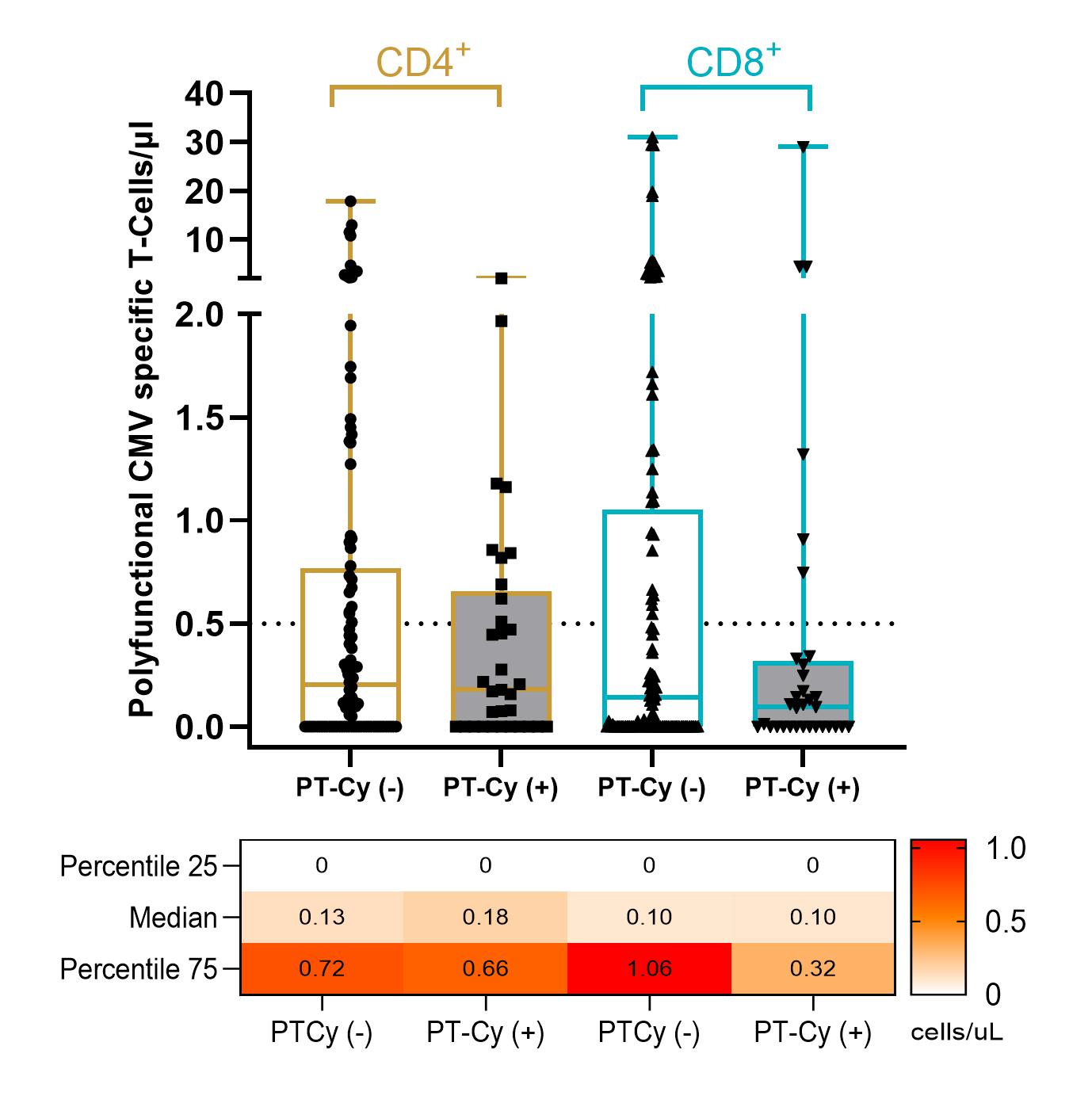
**Supplemental Figure 1**. Box-whisker graphs presenting median values with range after pp65 stimulation at day 90 after transplantation in patients according to whether they received post-transplant cyclophosphamide (PT-Cy). Absolute numbers of polyfunctional CD4^+^ (in brown outlines) and CD8^+^ cells (in teal outlines). Heatmap presenting 25^th^, 50^th^ and 75^th^ percentile for each category.


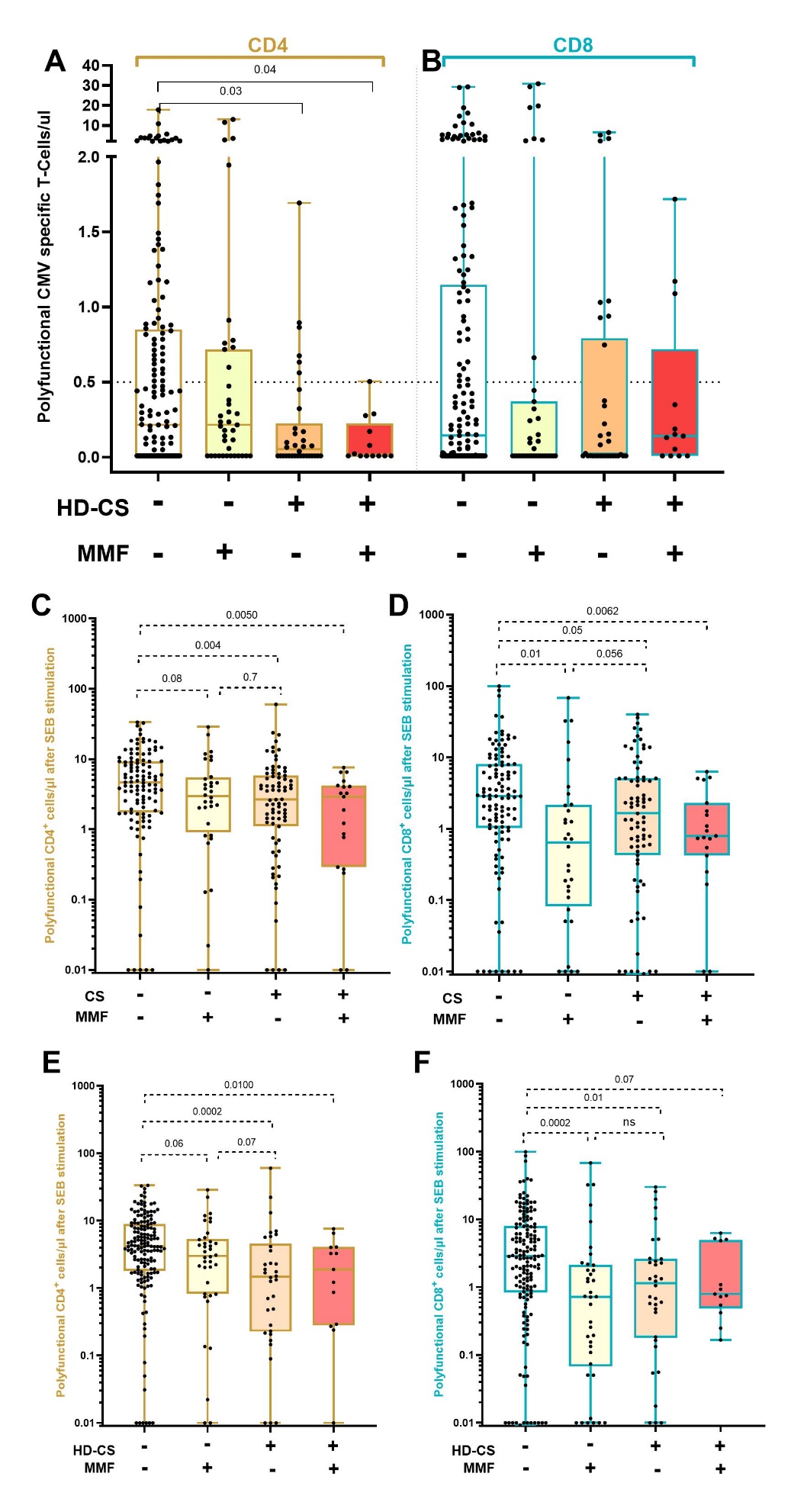


**Supplemental Figure 2**. Absolute numbers of polyfunctional CD4^+^ (in brown

outlines) and CD8^+^ cells (in teal outlines), in different drug categories. (A,B) – box-whisker graphs presenting median values with range after pp65 stimulation in patients receiving high dose (HD >0.5m/kg) of steroids (CS) and/or mycophenolate mofetil (MMF), (C-F) box-whisker graphs presenting median values with range after SEB stimulation in patients receiving any dose (C,D) or high dose (E,F) of corticosteroids and/or MMF).


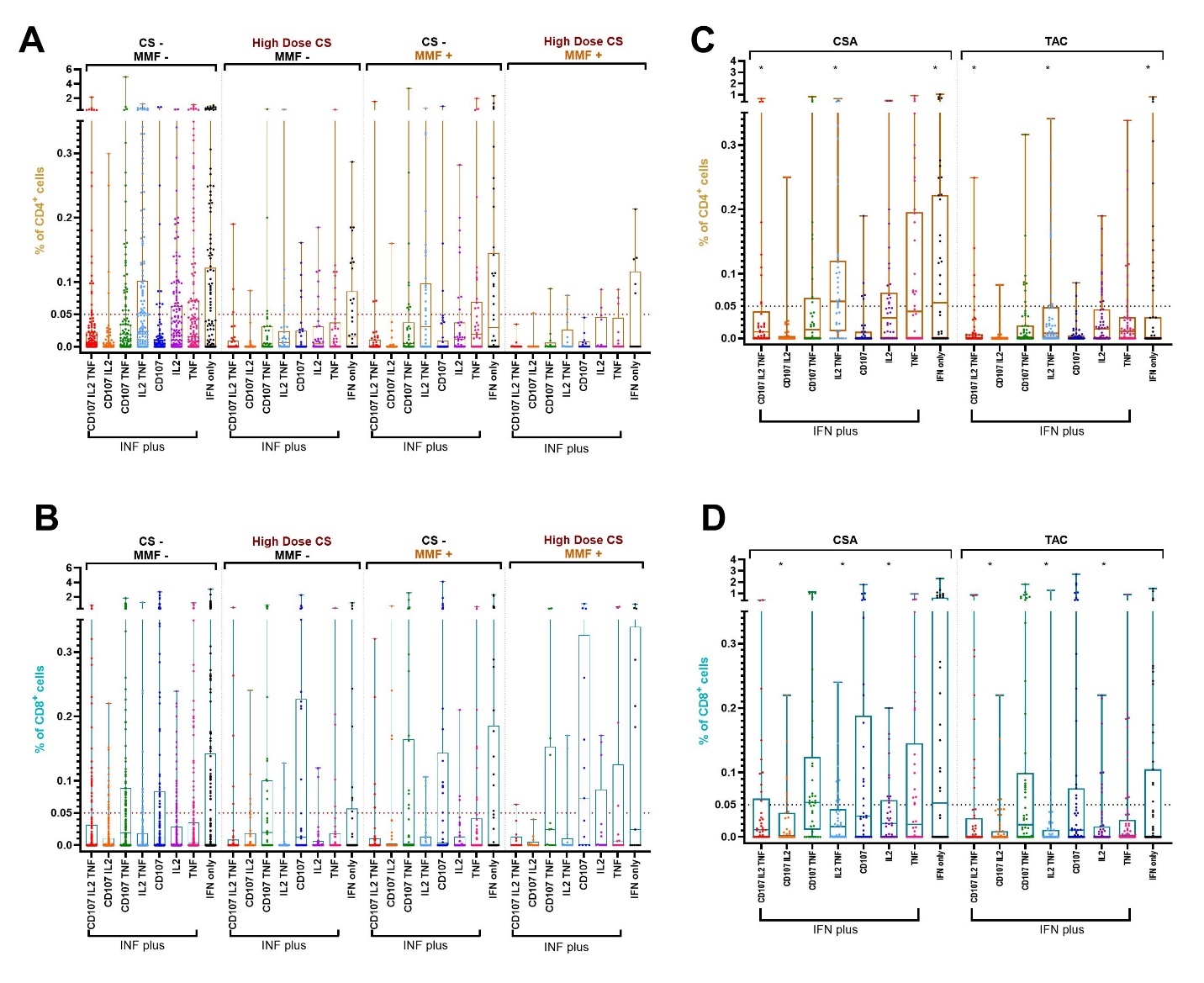


**Supplemental figure 3.** Frequencies of Polyfunctional (IFN-γ + ≥1) and Monofunctional (IFN-γ) CMV-Specific CD4⁺ (A,C) and CD8⁺(B,D) T Cells by Cytokine Profile and Immunosuppressive Regimen (A, B: Corticosteroids, MMF; C, D: Cyclosporine (CSA), Tacrolimus (TAC) among patients not exposed to MMF or steroids within 28 before sample collection

Distinct cytokine production patterns were observed between CSA and TAC recipients. Results with statistical significance are marked with *. Patients receiving CSA had higher frequencies of highly polyfunctional CMV-specific T cells, including IFN-γ^+^ TNFα^+^ IL-2^+^ 107a^+^ (p = 0.086 for CD4⁺, p = 0.05 for CD8⁺) and IFN-γ^+^ TNFα^+^ IL-2^+^ cells (p = 0.003 for CD4⁺), compared to those on TAC. Among CD8⁺ cells, the CSA group also showed a significantly higher proportion of IFN-γ^+^ IL-2⁺ producers (p = 0.01) and a trend toward increased IFN-γ⁺ TNFα⁺ cells (p = 0.06). Additionally, both CD4⁺ and CD8⁺ subsets in the CSA group exhibited higher frequencies of cells producing IFN-γ alone (p = 0.002 for CD4⁺, p = 0.09 for CD8⁺


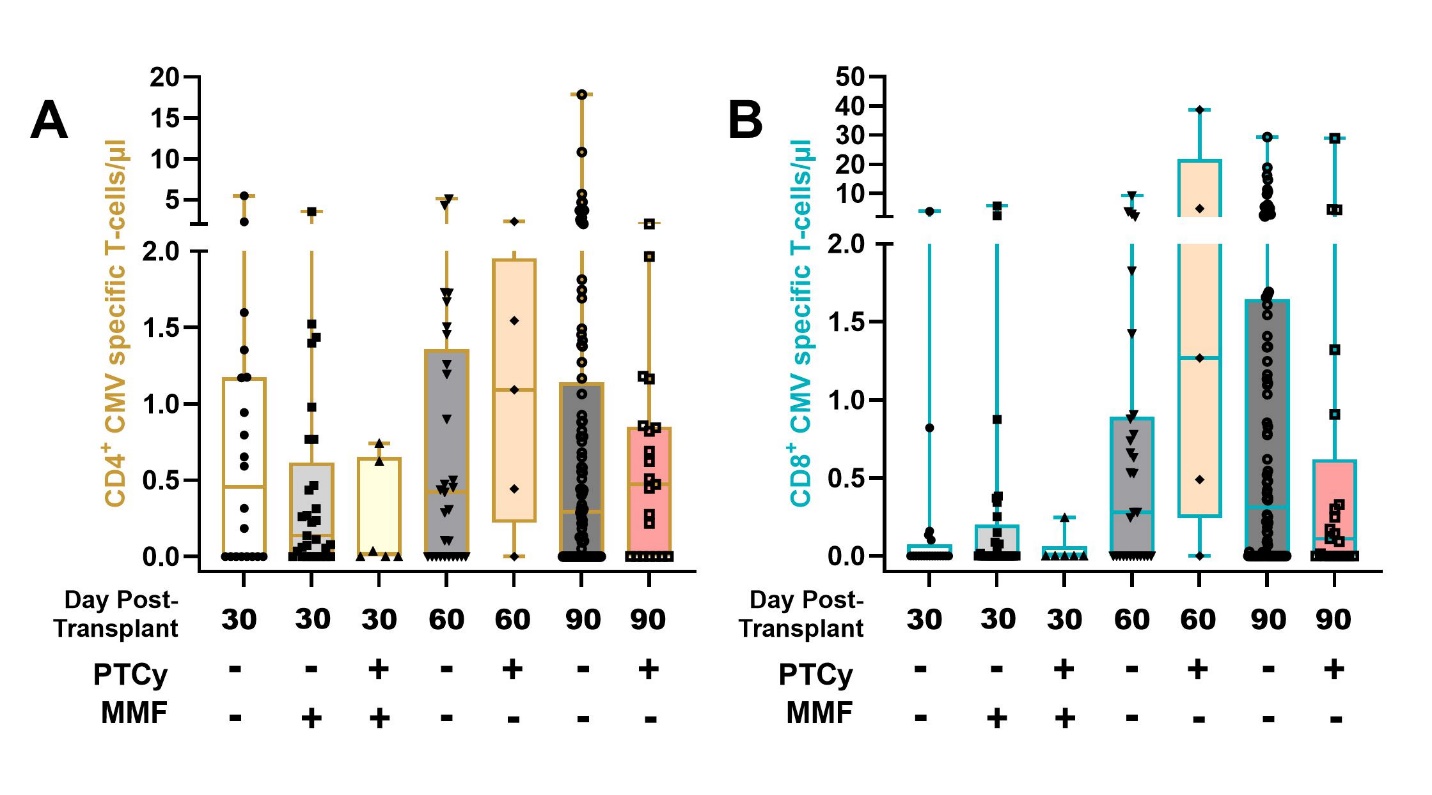


**Supplemental Figure 4**. Dynamics of CMV-specific immune reconstitution in patients not exposed to MMF and steroids at the time of sample collection stratified by PT-Cy administration, panel A – CD4^+^, panel B – CD8^+^.
